# Etiology, Clinical Features, and Diagnosis of Vulvar Lichen Sclerosus: A Scoping Review

**DOI:** 10.1155/2020/7480754

**Published:** 2020-04-21

**Authors:** Nilanchali Singh, Prafull Ghatage

**Affiliations:** Department of Gynecologic Oncology, Tom Baker Cancer Center, University of Calgary, Calgary, Alberta, Canada

## Abstract

*Objective*. Vulvar lichen sclerosus (VLS) is a chronic inflammatory disorder, which affects women of all ages. With numerous controversies as regards to the nomenclature, diagnosis and its association with neoplastic conditions, we decided to conduct a scoping review on this subject. *Data Source*. A review protocol was developed, and the Knowledge Resource Services website was used to do a search of articles pertaining to VLS with keywords “Vulvar,” “Vulval,” “diagnosis,” “lichen sclerosus et atrophicus,” “kraurosis,” “vulvar dystrophy,” and “Lichen Sclerosus”. *Study Selection*. The search was limited to published data from the last ten years, i.e., from July 2009 onwards and in the English language. A total of 338 articles pertaining to VLS were obtained. Older data were accessed if particular information was sought for. *Results & Conclusion*. The presentation is bimodal, i.e., one in prepubertal girls (average age: 7.6 years) and the other in peri- and postmenopausal women (average age: 52.6 years). However, many cases also present during reproductive years. Studies suggest a multifactorial origin as far as etiology is concerned, including a genetic, autoimmune, hormonal, and local infectious background. It affects the genital labial, perineal, and perianal areas and manifests as a patchy, thin, glistening, ivory-white area. Diagnosis is mainly based on clinical features. Biopsy is seldom required. It has been well established as a precursor lesion of dVIN and vulvar carcinoma.

## 1. Introduction

Vulvar lichen sclerosus (VLS) is one of the most common pathologies presenting to vulvar clinics. 13% of women present with symptomatic vulvar disease [1]. Prior to the 1975 International Society for the Study of Vulvovaginal Disease (ISSVD) classification, vague terminologies were used for vulvar Lichen sclerosis such as leukoplakia, kraurosis, and vulvar dystrophy. The current ISSVD classification includes this disease entity with vulvar dermatoses, which are nonneoplastic and noninfectious in nature. The literature shows evidence that they have a potential to transform into a vulvar intraepithelial neoplasia (VIN) and keratinizing vulvar carcinoma [2]. Also, VIN associated with LS is differentiated (dVIN); cancer seems to always be preceded by dVIN in the context of LS. The data, however, do not show whether there is clear evidence of a causal association between VLS and neoplasia or a mere coexistence [3]. It is also unclear whether a biopsy is mandatory for the initial diagnosis of VLS and whether treatment on the basis of symptomatology only can be started. With numerous controversies as regards to the nomenclature, diagnosis, and its association with neoplastic conditions, we decided to conduct a scoping review on this subject [4]. The focus of this review is on the etiopathogenesis, clinical features, and clinic-pathological diagnosis.

## 2. Methods

A review protocol was developed, and the Knowledge Resource Services (KRS) website was used to do a PubMed search of articles pertaining to VLS. Keywords used were “Vulvar,” “Vulval,” “diagnosis,” “lichen sclerosus et atrophicus,” “kraurosis,” “vulvar dystrophy,” “VIN,” “Cancer,” and “Lichen Sclerosus.” The search was limited to published data from the last ten years, i.e., from July 2009 onwards and in the English language. A total of 338 articles pertaining to VLS were obtained. Older data were accessed if particular information was sought for. For example, there are limited studies on nomenclature, prevalence, pathology, and testosterone usage in the past 10 years. It is not expectable to find papers about testosterone in the recent years, as it has been shown not to be useful and has severe adverse effects. All the articles were accessible in full text. Original articles and some review articles were given priority. In this review, individual data sources were not sought for, and a descriptive analysis was done. The data were summarized in form of a descriptive review.

## 3. Historical Perspective

Weir described vulvar or oral LS as “ichthyosis in 1875. Breisky described vulvar LS as Kraurosis Vulvae in 1885. The classic histopathology of LS was first described by Darier in 1892. It was, thereafter, labeled as lichen sclerosus et atrophicus, as it was considered to be an atrophic lesion. In 1976, it was labeled by Friedrich as a dystrophy rather than an atrophy; hence, the terminology “et atrophicus” was dropped. The ISSVD in 1976 discouraged the use of the term “Kraurosis” and “Leucoplakia,” and since then, the term Lichen Sclerosus has been used. VLS was first documented in the pediatric population in 1901.

Taussig in 1920 recommended surgery, i.e., vulvectomy, as the treatment of choice for kraurosis vulvae. However, it is no more recommended as a therapy for simple LS, without any neoplastic association. Retinoids were also used for its treatment in the past. The role of hormone therapy in its management started in the 1940s, when testosterone was being used. The reason for testosterone usage was that women suffering from LS were thought to be testosterone deficient related to a testosterone dehydrogenase deficiency at the skin. However, with the advent of local steroid therapy in 1961, the role of testosterone diminished to an extent that it is no more used. After usage of various steroid compositions, the fluorinated superpotent steroids like clobetasol were the recommended in 1980s. HLA serotyping and causal associations with infections like *Borrelia* were also reported in this decade.

## 4. Epidemiology

Though the exact prevalence of VLS is not known, the rate of histologically proven VLS is 1.7% in general gynecology practice, according to a study. 54% of these women were postmenopausal (mean age 52.6 years) [5]. The prevalence of LS is probably underestimated since a third of cases are asymptomatic. The presentation is bimodal, i.e., one in prepubertal girls (average age: 7.6 years) and the other in peri- and postmenopausal women (average age: 52.6 years). However, many cases also present during reproductive years [6, 7]. The prevalence of VLS in postmenopausal women is reported to be 1 : 30. The incidence of VLS is reportedly been rising. According to a Dutch Registry Data, it has been found to increase from 7.4 per 100,000 woman-years in 1991 to 14.6 per 100,000 woman-years in 2011 [1].

## 5. Etiology and Risk Factors

The etiology of LS remains unknown, but several mechanisms have been studied for this noncontagious disease. Studies suggest a multifactorial origin as far as etiology is concerned, including a genetic, autoimmune, hormonal, and local and infectious background. The risk factors are mentioned in Table 1.

### 5.1. Genetic Predisposition

Genetic association to LS has been shown in family and twin studies [8]. Sherman et al. studied 1052 women with LS (80% histologically confirmed) using family clustering and reported that 126 (12%) women had a positive family history of the condition [9].

### 5.2. Autoimmunity

There is a strong association between LS and autoimmune disease in adults [10]. Some specific antibodies have been reportedly, associated with VLS. Furthermore, some autoimmune diseases like diabetes mellitus type 1, autoimmune thyroiditis, psoriasis, and vitiligo have been associated with the disease.

### 5.3. Infectious Etiology

Infections such as with *Borrelia burgdorferi*, causative agent of Lyme disease, have been implicated, but the evidence is contradictory [11]. Viral etiology associated with HPV and hepatitis C has also been suggested as an etiology.

### 5.4. Local Causes

A well-known manifestation of VLS is the Koebner phenomenon. It is described as the occurrence of lesions at sites of injured or traumatized skin due to scratching or sexual activity. Thus, repeated trauma and irritation to the area may act as a precipitating factor for the disease [12]. Radiation has also been implicated as one of the causal factors.

### 5.5. Hormonal Etiology

The bimodal distribution of the disease in prepubertal and postmenopausal women suggests its association with low estrogen levels as in these women. However, studies proving this theory are lacking. Considering the possibility of testosterone deficiency in local tissues of women with VLS, testosterone has been used for treating the condition in past. To date, there is little evidence to this theory too. Increased risk of VLS in Turner syndrome, but lack of variation with use of HRT and contraceptive pills, points to controversial role of hormones. The role of hormones has been put aside in recent years.

## 6. Clinical Characteristics

### 6.1. Signs and Symptoms

**Figure 1:**
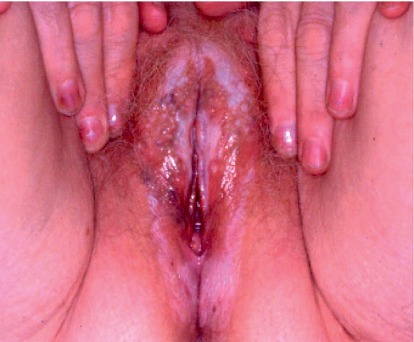
White patches seen in vulvar lichen sclerosus.

**Figure 2:**
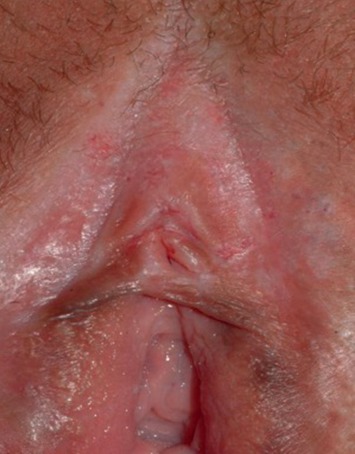
Early stage of vulvar lichen sclerosus with glistening white skin.

**Figure 3:**
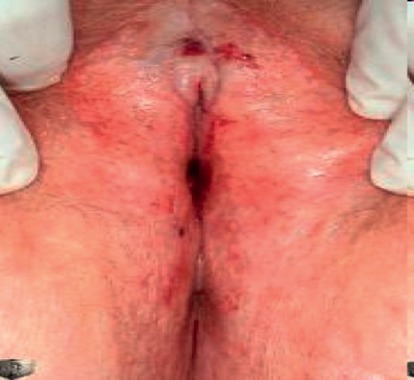
Late stage of vulvar lichen sclerosus with loss of labia minora and ulcerative lesions with hemorrhage seen on vulva.

Vulvar lichen sclerosus (LS) is a chronic, progressive, inflammatory, nonneoplastic epithelial disease. It affects the genital labial, perineal, and perianal areas and manifests as a patchy, thin, glistening, ivory-white area (Figures 1 and 2). In 20% of patients, other areas are involved such as the upper trunk, axillae, buttocks, and lateral thigh. It does not affect the vagina and cervix. It usually presents with progressive pruritus, but it may also present with burning, dyspareunia, apareunia, anorgasmia, dysuria, or genital bleeding. Constipation and painful defecation may be a feature in case of perianal involvement but is rare in adult women. It may be asymptomatic in some women. It also leads to significant discomfort and psychological distress in many women. Examination may reveal an sore, bright and red vulva in initial cases. Hemorrhagic bullae may be also present in some cases, which can be confused with other vulvar ulcerative disease [12]. With time, the vulva becomes pale with atrophic changes leading to loss of labia minora, burying of the clitoris, obstruction of urinary outflow, reduction of the vaginal introitus, and fourchette adhesions (Figure 3). Chronic scratching due to pruritus can result in subepithelial hemorrhage. Perianal involvement can lead to the classic “figure of eight” shape of the lesion. Clinical features of VLS are summarized in Table 2.

The Delphi exercise for the signs and symptoms of the severity of adult lichen sclerosus has been suggested. However, the method of measurement of the signs and symptoms was not reached. Hence, further research is advised on this severity scale [13].

### 6.2. Complications

Lichen sclerosis can lead to skin cracks and bleeding, leading to painful, sore areas with secondary infections. It may lead to scarring and narrowing of the vaginal introitus, making coitus almost impossible. With severe scarring and deformity, urinary retention, and anal stenosis, obstruction and constipation may occur. It may also sometimes transform to a premalignant or a malignant lesion.

### 6.3. Comorbidities

VLS is associated with other immune disorders such as morphea or localized scleroderma, systemic sclerosis, Hashimoto's thyroiditis, rheumatoid arthritis, psoriasis, diabetes mellitus type 1, and alopecia areata [14]. In a series of 211 patients with confirmed LS, 29.9% had thyroid disease, which is much higher than in the general population [15]. In a series of 472 patients with morphea, LS was more frequent as indicated by an odds ratio of 18.1 [16]. The prevalence of psoriasis in LS women (7.5%) is higher than in the general population; thus, a po6ssible association between LS and psoriasis has also been reported. Oral lichen planus (LP) and vulvar LS may coexist [17].

In a series of 308 women with LS seen at a vulvar clinic, overactive bladder was seen in 15.3%, stress urinary incontinence in 27.9%, constipation in 32.5%, irritable bowel syndrome in 19.5%, thyroid dysfunction in 33.1%, fibromyalgia in 9.1%, temporomandibular joint disorder in 13.0%, and vulvar pain in 83.1% [18].

Higher prevalence of overweight or obesity as well as of hypertension in LS patients in comparison with the general Italian population has been reported in a study. Accordingly, a possible metabolic contribution in the multifactorial etiopathogenesis of LS was assumed [19].

### 6.4. Associated Premalignant and Malignant Conditions

Vulvar lichen sclerosus has a nonnegligible risk of neoplastic transformation to squamous cell carcinoma and therefore requires a vigilant and lifelong follow-up in all patients, particularly in elderly women. Early detection of premalignant lesions is necessary to reduce the risk of vulvar cancer, which may be achieved by careful screening in these women. Vulvar squamous cell carcinoma is seen in up to 3.5–7% of women with VLS [1, 7, 8, 20–22]. Topical corticosteroid use in women with VLS can achieve disease remission and reduce the risk of malignant transformation [23]. Recent data show that the risk is low in women appropriately treated and under follow-up. Longer duration of symptoms and loss of vulvar architecture increase the risk of cancer. According to a large study comprising of 976 cases, the neoplasia incidence risk was 3.5% and the neoplasia incidence rate was 8.1 per 1,000 person-years [20]. The cumulative probability of progression to neoplasia increased from 1.2% at 24 months to 36.8% at 300 months. The median progression-free survival was significantly shorter in older women (≥70 years) when compared with that in younger women (*p*=0.003). The study suggested that around 1% of patients diagnosed with VLS could develop a neoplasia each year. The Kaplan–Meyer survival curve for the study population showed that the cumulative probability of progression to neoplasia increases from approximately 1% at 2-year follow-up to nearly 37% at 25-year follow-up. It, therefore, mandates a lifelong follow-up [20]. Women with LS-associated vulvar cancer are significantly older than women with LS alone, and SCH is independently associated with vulvar carcinoma.

Differentiated vulvar intraepithelial neoplasia (dVIN) is a rare type of vulvar intraepithelial neoplasia (VIN), which is usually seen in older women and is associated with lichen sclerosus. Although most cases of VIN will not develop into cancer, it is not possible to tell which one will. dVIN associated with lichen sclerosus is more likely to be associated with a squamous cell carcinoma of the vulva than usual type VIN. Furthermore, it has a higher recurrence rate and decreased disease-specific survival from invasive squamous cell carcinoma. dVIN is associated with LS and that one definitely is a risk factor for cancer. It is not likely that LS develops directly to cancer. This pathway of malignancy is HPV independent. VIN has a high risk of progression, which is much higher than HSIL.

### 6.5. Psychosexual Impact

VLS does impact the psychosexual health of women. According to a study, women with VLS have a lower score on the 7-item Female Genital Self-Image Scale compared with that of healthy controls. These women have increased rates of dyspareunia and concerns of loss of relationships, with desires to regain the experience of intimacy and sexual enjoyment as a major motivation for surgical interventions for their condition [24]. This negative influence of vulvar lichen sclerosus on female genital self-image has a correlation to sexual arousal, orgasm, and satisfaction rates. They also report significantly less frequent sexual activity, lower satisfaction with sexual activity, depression, and poor quality of life [25].

A third of patients experience impairment of health-related quality of life. To improve dermatological care, enhancement of doctor-patient communication, information provision, and organization is recommended [26].

### 6.6. Juvenile Vulvar Lichen Sclerosus

Juvenile vulvar lichen sclerosus is seen in around 1 : 900 children. A study of 327 patients with LS showed a mean age of onset of disease at 5.4 years in girls. Delayed diagnosis is common in girls with LS, with an average duration until diagnosis of 1 to 1.6 years [27]. Juvenile VLS may be asymptomatic but usually manifests as pruritus, pain, burning sensation in the perineum, constipation, or urinary symptoms, as seen in adults. The pathognomonic characteristic is the presence of a depigmented lesion in the shape of an “8”, involving the anogenital region. Lesions are white, flat-topped papules, thin plaques, or atrophic patches initially. Purpura is a hallmark feature of vulvar LS. Hyperpigmentation, erosions, and ulceration can occur. Secondary constipation is also a common complication, occurring in 67% of girls with anogenital LS. Young girls will withhold stooling due to the pain; subsequent management can be quite difficult, with habits and symptoms persisting even after effective treatment of the LS. Constipation and obstipation is a feature of juvenile LS, which is uncommon in adults. Due to the nature of the symptoms, there may be a suspicion for child abuse. LS is often misdiagnosed as sexual abuse, but if they coexist, it worsens the LS (isomorphic or Koebner phenomenon).

Due to the chronicity of inflammation leading to scarring, the orifices in affected area may obliterate. The disease can relapse and become a lifelong condition. In a prospective case series in which 12 girls were followed from prepubertal years until adolescence, 25% had complete remission, whereas 75% remained symptomatic with clinical signs of the disease as adolescents [28]. Even though they were diagnosed early and received treatment, childhood onset LS may be complicated by distortion of vulvar architecture. Other complications include a 5% lifetime risk of developing squamous cell carcinoma. Vulvar melanoma has also been reported in a case of juvenile VLS [29]. Juvenile VLS requires intensive treatment. It cannot be treated with menarche and those scars can be irreversible.

## 7. Histocytochemistry

### 7.1. Histopathology

**Figure 4:**
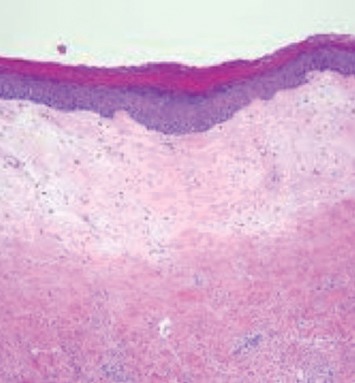
Thin epithelium with loss of rete edges, hyperkeratosis, fibrin deposition, and loss of vascularity and chronic inflammatory cell infiltrate of lymphocytes in deeper layer on histopathology.

VLS is characterized by skin changes of vulva. Characteristically, vaginal involvement is not seen. Microscopically, there is severe hyperkeratosis; thin epidermis, loss of rete pegs, basal cell degeneration, homogenized band of dense fibrosis at papillary dermis, upper dermal edema, and often band-like chronic inflammation around vessels (particularly eosinophils) (Figure 4). In early stages, findings are subtle and often more prominent in adnexal structures than in interfollicular skin. Adnexal structures show acanthosis, luminal hyperkeratosis, and hypergranulosis with or without dystrophic hair and basement membrane thickening. Early dermal changes are homogenized collagen and wide ectatic capillaries in dermal papillae immediately beneath basement membrane. Lymphocytic infiltrate can be sparse or dense. The proposed minimal histologic criteria are vacuolar interface reaction pattern in conjunction with dermal sclerosis (homogenized and hyalinized eosinophilic collagen bundles) of any thickness intervening between inflammatory infiltrate and epithelium or vessel walls [30]. The typical finding are often absent, especially after steroid treatment.

### 7.2. Immunocytochemistry

There is a controversial role of immune response in etiopathogenesis of vulvar lichen sclerosus. One article reported eosinophilic spongiosis in VLS [31]. Galectin-7, a keratinocyte belonging to the galectin family and was initially described as a marker of epithelial differentiation, due to its expression in the stratified epithelium of various tissues, has been studied for its role in VLS. According to a study, expression of galectin-7 was significantly upregulated in VLS tissues [32]. In addition, galectin-7 significantly inhibited the viability of dermal fibroblasts and stimulated the production of collagen. Thus galectin-7 may be a potential drug target for the treatment of patients with VLS. One study showed an increase in the deposition of abnormal collagen type V (COLV) with a correlation to the extracellular matrix 1 and elastic fibers suggesting that COLV may be a trigger in the pathogenesis of lichen sclerosus [33]. Various genes and molecules such as p53, CK-5, COX-2, and osteopontin have been studied in women with VLS, with variable degrees of association. There are also reports of autoimmunity to basement membrane zone proteins in childhood and adult VLS.

### 7.3. Role of Testosterone Receptors and Levels

Older studies have shown that serum levels of dihydrotestosterone, free testosterone, and androstenedione were significantly decreased in patients with untreated vulvar lichen sclerosus [34]. There are studies, which claim that the levels of testosterone should be measured in women before prescribing them local testosterone therapy, to avoid virilization effects in normoandrogenic women [35]. The Cochrane review 2011 did not find any use of topical testosterone in the treatment of vulvar lichen sclerosus [36]. Presence of androgen receptors (AR) has been shown to influence the disease presentation in VLS. A study suggested that AR positive patients are asymptomatic and AR negative ones present with symptoms [37]. One study has found that topical testosterone worsens the symptoms in comparison with placebo [38]. Hence, the role of testosterone and its usage in treatment is completely refuted in the current scenario.

## 8. Genetic Profile

Recurrent germ-line variants in four genes have been identified as likely to be deleterious to proper protein function in cases of VLS. The genes with variants include *CD177* (neutrophil activation), *CD200* (inhibitory signal to macrophages), *ANKRD18A* (ankyrin repeat protein, epigenetic regulation), and *LATS2* (corepressor of androgen signaling). Although clinical significance of these genomic alterations is uncertain at this time, previous research suggests that neutrophil activation and macrophage inhibition may be related to granulomatous and autoimmune diseases, whereas ankyrin repeat protein and corepressor androgen signaling have been linked to tumor suppressor activities. Future research should focus on identifying whether these similarities are present in other families with vulvar LS to better understand the pathophysiology of this condition and guide treatment [39].

Recently, epigenetic pathways have been implicated as causative or accelerant agents of disease, particularly miR-155, downstream targets of ECM1, galectin-7, p53, and epigenetic modifications to CDKN2A [40].

## 9. Diagnosis

### 9.1. Diagnostic Workup

The diagnosis of LS is usually clinical. Careful history taking and clinical examination are the mainstays of diagnosis. Late presentation and lack of recognition of symptoms may lead to diagnostic delay, at times. Women presenting with vulvar pruritus and pain should be examined by a health professional with expertise in vulvar skin disorders. While women should be encouraged to examine their vulva for any changes, this may be difficult or impossible in the elderly women with comorbidities. Therefore, careful inspection of the external and internal genitalia is recommended during every gynecologic consultation, particularly in postmenopausal women. Correct and timely diagnosis and early aggressive treatment are crucial to prevent complications.

Since the diagnosis of LS is usually clinical, biopsy is reserved for cases if there is a doubt in diagnosis, a suspicion for preneoplastic and neoplastic change such as vulvar intraepithelial neoplasia and vulvar cancer, resistance to adequate treatment, or atypical extragenital presentations. Biopsy should be performed from interface between normal and abnormal areas. Biopsy should also be performed of the hyperkeratotic areas and erosions that do not improve with treatment or sites with altered pigmentation [41].

The role of dermoscopy in vulvar LS has been sparsely studied and requires further evaluation. Smaller studies accessing the role of dermoscopy in monitoring the treatment response have been there, and they suggest its usefulness [42] The researchers have described that VLS exhibits characteristic dermoscopic patterns that can aid in its clinical diagnosis. The lesions exhibit sparse dotted vessels; patchy, structureless areas, whitish to white-yellow to pink-whitish color over a diffuse whitish background; grey-blue dots, usually with a characteristic peppered arrangement, corresponding to dermal melanophages; comedo-like openings and scales; as well as peculiar structures like ice slivers [42].

Assessment should also include workup for autoimmune diseases such as type 1 diabetes mellitus, thyroid disease, scleroderma, and rheumatoid arthritis as there is an association between VLS and autoimmune diseases. The assessment should be clinical, and investigations should be performed when indicated.

### 9.2. Differential Diagnosis

VLS is commonly misdiagnosed as *Candida albicans* vulvitis or thrush or postmenopausal atrophy. This may lead to diagnostic delay of up to five years [19]. The differentials also include lichen planus, localized scleroderma, leukoplakia, and vitiligo and immunobullous disorders such as cicatricial pemphigoid, cutaneous patch of Lyme's disease and vulvar intraepithelial neoplasia (VIN). Ecchymotic and bleeding lesions may lead to suspicion of sexual abuse in children. Squamous cell hyperplasia, which increases the risk of vulvar malignancy, may be present in acanthotic areas of VLS [41].

## 10. Conclusion

The presentation of lichen sclerosus is bimodal in prepubertal girls and in peri- and postmenopausal women. It affects the genital labial, perineal, and perianal areas and manifests as a patchy, thin, glistening, ivory-white area. Diagnosis is mainly based on clinical features. Biopsy is seldom required. Studies suggest a multifactorial origin as far as etiology is concerned, including a genetic, autoimmune, hormonal, and local and infectious background.

## Figures and Tables

**Table 1:** Etiology and risk factors of vulvar lichen sclerosus.

Genetic predisposition
Autoimmunity
Infectious etiology (*Borrelia burgdorferi*, human papilloma virus, hepatitis C)—doubtful
Local causes (*Koebner* phenomenon)
Hormonal etiology (low estrogen levels and testosterone deficiency)

**Table 2:** Clinical features of vulvar lichen sclerosus.

Signs and symptoms

Asymptomatic
Chronic pruritus
Dyspareunia or apareunia
Anorgasmia
Dysuria
Genital bleeding
Constipation
Painful defecation
Pale lesion with atrophic changes in vulva
Loss of labia minora
Burying of the clitoris
Obstruction of urinary flow
Reduction of the vaginal introitus
Fourchette adhesions
Subepithelial hemorrhage due to pruritis
Classic “figure of eight” shape of the lesion

Comorbidities
Morphea or localized scleroderma
Systemic sclerosis
Hashimoto's thyroiditis
Rheumatoid arthritis
Psoriasis
Diabetes mellitus type 1
Alopecia areata
Overactive bladder
Stress urinary incontinence
Irritable bowel syndrome
Fibromyalgia
Temporomandibular joint disorder

Associated Malignancy
Neoplastic transformation to squamous cell carcinoma
Association with melanoma reported

Pschosexual Impact
Concerns of loss of relationships
Desires to regain intimacy and sexual enjoyment
Negative influence on female genital self-image
Negative correlation with sexual arousal, orgasm, and satisfaction rates
Less frequent sexual activity

## Additional Points

*Highlights.* The presentation of vulvar lichen sclerosus is bimodal, i.e., one in prepubertal girls (average age: 7.6 years) and the other in peri- and postmenopausal women (average age: 52.6 years). Most cases also present during reproductive years.

  The current ISSVD classification includes this disease entity with vulvar dermatoses, which are nonneoplastic and noninfectious in nature.
  Lichen sclerosus has a multifactorial origin. The etiology can be genetic, autoimmune, hormonal, or local infectious in nature.
  It manifests as a patchy, thin, glistening, white area involving labia and perineum. Diagnosis is mainly based on clinical features. Biopsy is seldom required.
  Vulvar lichen sclerosus has a nonnegligible risk of neoplastic transformation to squamous cell carcinoma and therefore requires a vigilant and lifelong follow-up in all patients, particularly in elderly women.
  The diagnosis of LS is usually clinical. Careful history taking and clinical examination are the mainstays of diagnosis.
  Biopsy is reserved for cases if there is a doubt in diagnosis, a suspicion for preneoplastic and neoplastic change such as vulvar intraepithelial neoplasia and vulvar cancer, resistance to adequate treatment, or atypical extragenital presentations.
